# Performance using a visuo-haptic surgical simulator is affected by age

**DOI:** 10.1186/s41077-026-00442-x

**Published:** 2026-06-17

**Authors:** Mara Coduri, Chiara Saporetti, Michele Minuto, Maura Casadio, Serena Ricci

**Affiliations:** 1 Department of Informatics, Bioengineering, Robotics and Systems Engineering, University of Genoa, Genoa, Italy; 2 Simulation and Advanced Education Center, University of Genoa, Genoa, Italy; 3 Department of Surgical Sciences and Integrated Diagnostics, University of Genoa, Genoa, Italy

**Keywords:** Manual skills, Surgical training, Visuo-haptic simulator

## Abstract

**Background:**

Surgeons need to master many manual skills, which potentially decrease with age. Yet, there is no standardized approach to objectively assess how aging affects basic surgical performance and skills training. In this context, simulation offers a promising solution, being widely used to train and test novice clinicians. This study aims to evaluate the impact of age on performance using a visuo-haptic surgical simulator.

**Methods:**

A total of 39 participants were divided in two groups: 20 young (20–40 years) and 19 older adults (50–70 years). All participants completed multiple repetitions of three basic surgical tasks using a custom visuo-haptic simulator: incision, an adapted version of the needle threading task, and suturing. Performance metrics such as errors, trajectory, and task duration were compared between groups and across repetitions.

**Results:**

Younger participants outperformed older ones in all tasks. Specifically, they cut more tissue, made fewer errors in the dexterity task, and sutured faster. Both groups partially improved with practice, although older subjects improved less than younger ones.

**Conclusions:**

Altogether these results suggest that age significantly affects performance in basic surgical tasks; moreover, older subjects might benefit from periodic training sessions to maintain their skills. In this regard, visuo-haptic simulation can serve as an effective training and evaluation tool to assess and counteract the effects of aging on surgical ability.

## Background

Surgical performance is a complex construct that combines both technical and non-technical competencies, including decision-making and communication. Regarding the technical skills, surgeons need to master a wide range of visual, perceptual and fine motor skills to ensure safe and effective surgical procedures [1].

Several studies have demonstrated that fine motor skills decrease with aging; however, despite this decline, learning capabilities are maintained [2], suggesting that practice and training can compensate for fine motor performance worsening [2].

In the specific context of surgery, age-related decrease in fine motor control and psychomotor speed can negatively impact surgical performance, even though experience may slow down this process [3, 4]. Various studies have focused on understanding the relationship between surgical skills and age, with the goal of defining the appropriate retirement age. However, these studies investigated post-operative complications, peer evaluations or physical and cognitive tests [3, 5], rather than changes in surgical ability. Indeed, there is growing consensus on the need for objective tools to evaluate residual skills in aging surgeons [4], as currently no standardized approach for assessing age-related changes in surgical skills and clinical judgment exists [6]. This might be due to two reasons: (i) procedures can be highly variable, thus making it hard to quantify technical skills on site; (ii) they require to equip the surgeons and/or the operating table with sensors or other systems to collect behavioral data.

Simulation-based training has emerged as a valuable strategy to maintain surgical proficiency, enabling repeated and targeted practice without clinical risk [1, 7]. This methodology has been widely used as a training and evaluation tool for students and novice surgeons. Furthermore, simulators might allow early detection of performance deterioration and can support the development of personalized retraining protocols. Thus, incorporating simulation into continuous medical education may be key to ensuring patient safety and extending surgeons’ safe practice life span [8]. Interestingly, even though there is general agreement on the validity of simulators for assessing learners’ competence [7], studies aimed at assessing the performance of aging surgeons do not use simulators as testing tools [3].

Within this framework, the present study investigates whether younger and older individuals exhibit differences in performance while training basic surgical skills using a custom visuo-haptic surgical simulator [9, 10]. Specifically, we isolated and examined the technical motor components underlying surgical task execution, with the ultimate goal of exploring how healthy aging influences the technical and kinematic execution of surgical gestures. The originality of this work lies in the application of simulation not only as a training platform, but also as a controlled environment to analyze age-related variability in fine motor performance.

## Methods

The simulator consists of a haptic device combined with a realistic visuo-haptic model of skin. It implements three exercises: incision, suture, and an adapted version of the needle threading task designed to improve dexterity, depth perception and hand-eye coordination.

As haptic device, we selected the Geomagic Touch (3D Systems, USA; [11]), which is a device commonly used in medical and surgical simulation [12, 13]. It can provide force feedback on the users’ hand, thus allowing to feel virtual objects and producing touch sensations as the user manipulates 3D objects.

The Virtual Environment was developed using SOFA framework (version 20.12.02; [14]) along with two key plugins: the Geomagic plugin to interface the simulation with the haptic device, enabling real-time force feedback; SofaPython3 to script within SOFA using a Python interpreter. To ensure reproducibility of the proposed system, Appendix A contains detailed hardware specifications, software parameters, and task configuration description.

### Surgical tasks

Three surgical tasks have been implemented targeting incision’s ability, hand-eye coordination, and suturing.

**Fig. 1:**
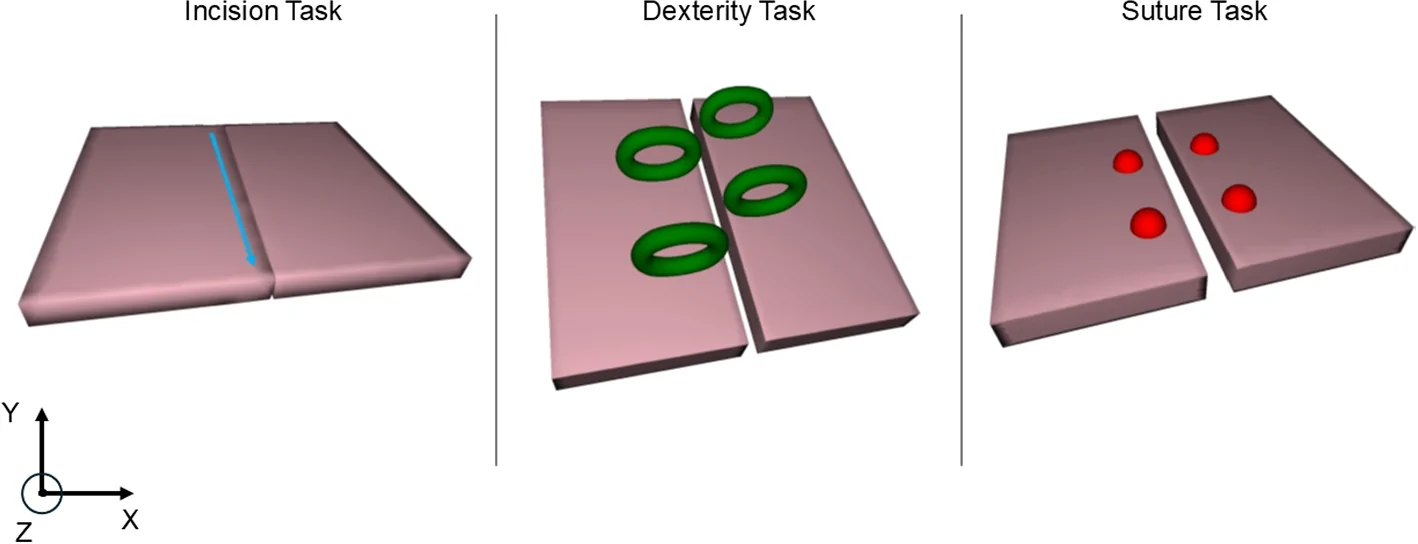
Virtual representation of the surgical task. Left: incision virtual task; Center: dexterity virtual task; Right: Suture virtual task

Incision Task trains the user to perform incisions along a defined path. The virtual model comprises two adjacent skin patches, each measuring 7 *× * 11 *× * 1.5 cm. The top layers of the patches are interconnected by invisible springs. Specifically, eight box-shaped regions of interest (ROIs), each measuring 0.7 *× * 1.5 *× * 2 cm, are defined along the top edges to generate localized spring force fields. The bottom layers are separated by a gap of 0.5 cm. A visible cutting line, extending 11 cm across the surface (Fig. 1), serves as a guide for the user during the procedure. During the simulation, the user manipulates a virtual scalpel using an adapted Geomagic Touch end effector (see Appendix A, for further information on the end-efectors 3D models). Upon contact between the scalpel blade and the skin surface along the cutting line, the invisible springs within the incision area rupture, realistically simulating tissue separation.

Dexterity Task is an adapted version of the needle threading task. It trains depth perception, hand-eye coordination, and precise manipulation skills. The virtual environment consists of four toroids whose center is located 1.5 cm above two adjacent skin patches (10 x 16 x 2.5 cm); both the patches and the toroids provide haptic feedback (Fig. 1). The user controls a capsule-shaped object using the Geomagic Touch. The task requires the subject to move the capsule through the toroids without touching them, starting with the farthest and progressing to the closest. If the capsule touches a toroid, the toroid turns red, providing visual feedback about the collision.

Suture Task requires users to connect adjacent skin patches using a suture needle. Two parallel skin patches (10 x 16 x 2.5 cm) are combined with four red spheres marking the locations where the needle should pierce the skin (Fig. 1). Each sphere corresponds to a boxROI (interactable region of interest) 4 x 4 x 3 cm that identifies the underlying skin indices. The user controls a virtual suture needle using the Geomagic Touch (i.e., the adapted needle holder handle, see Appendix A). When the needle pierces the skin in correspondence of a sphere and with the correct orientation, the sphere turns green and springs are generated between the skin and the needle. These springs allow the needle to pull the skin simulating a perceived elastic force. Springs are also created between the two skin patches when their spheres are pierced in sequence, simulating wound closure.

### Participants

We tested two groups of subjects: 20 young subjects (20-40 years; mean ± STD: 29.6 ± 5.4 years, 12 female, 2 left handed) and 19 older subjects (50-70 years; mean ± STD: 59.8 ± 5.2 years, 10 female, 4 left handed). These ranges were selected to avoid age overlap and to maximize separation between groups. The younger range reflects stable motor performance, while the older range includes individuals at risk for age-related motor decrease [15]. The upper age limit was set at 70 years because this approximates the typical retirement age for surgeons. We considered this to represent the oldest age of practical interest for evaluating simulator performance. The main objective of the study was to investigate the effect of age using a visuo-haptic surgical simulator, independently of prior surgical expertise; therefore, participants’ medical and surgical experience was not used as an inclusion criterion. Nevertheless, 5 out of 20 participants in the younger group and 7 out of 19 participants in the older group reported at least three years of surgical experience and were actively practicing at the time of the study.

### Experimental design

**Fig. 2:**
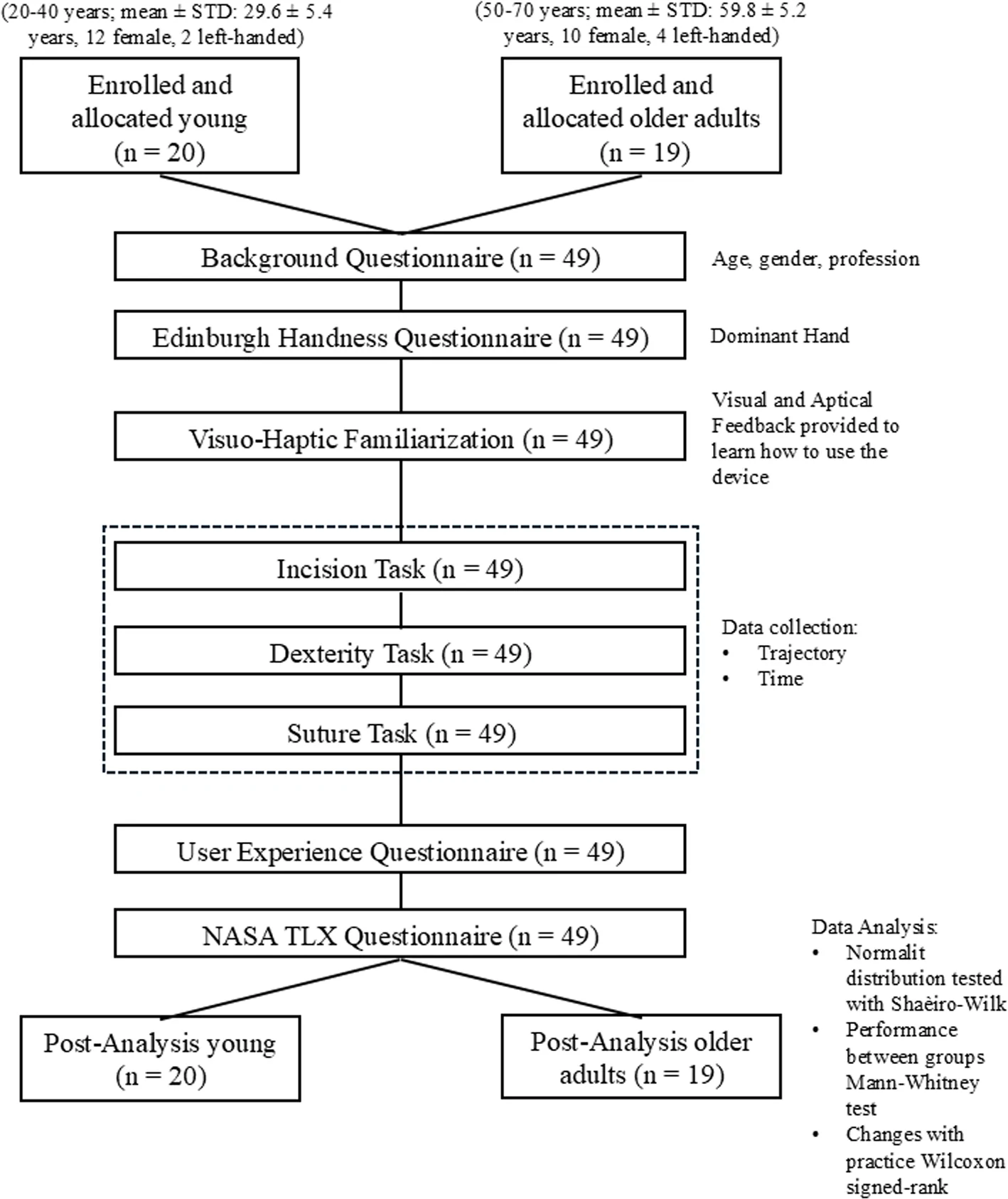
CONSORT-style flow diagram of participants through the visuo-haptic simulation study, structured by age groups

A CONSORT-style flow diagram is provided (Fig. 2) to illustrate participant recruitment and allocation throughout the study phases, in line with simulation-based research reporting guidelines [16].

The experiment lasted approximately 40 minutes, was conducted individually, under the supervision of an experimenter, and was divided into the following four phases:

#### Pre-questionnaires

Before the practical session, participants were asked to fill out a demographic questionnaire to gather information about age, gender and professional background, as well as the Edinburgh Handness Questionnaire (EHQ, [17]), a validated self-report instrument used to assess hand dominance. The questionnaire evaluates hand preference across a range of everyday activities, generating a laterality index that classifies individuals as left-handed, right-handed, or ambidextrous.

#### Familiarization

To account for any possible bias due to the technology used, we structured a familiarization phase ensuring that all the participants could use the system without any difficulty. The familiarization phase introduced users to the Geomagic Touch device and the screen-based virtual environment. It included three exercises to develop an understanding of haptic feedback and 3D navigation using the end-effector: following a straight line in space; touching and pressing a cube showed on the screen; touching and pressing a sphere. Each exercise was performed under the supervision of an experimenter who could provide instructions on how to use the device. Participants were allowed to repeat the familiarization exercises until they reported feeling comfortable with the device. No performance feedback related to the experimental tasks was provided during this phase.

#### Surgical tasks

After the familiarization, participants were asked to perform multiple repetitions of the three tasks described above in the order that follows: 6 repetitions of the incision task (the patches have three different orientation in the z axis), 6 repetitions of the dexterity task, and 8 sutures.

#### Post questionnaires

After completing the tasks, users were asked to complete two validated questionnaires: (i) Short-User Experience Questionnaire (UEQ; [18]) to evaluate the overall usability and experience with the system; (ii) NASA Task Load Index (NASA TLX; [19]) to assess subjective workload perception during the tasks.

UEQ measures the user’s subjective impression during interaction with the simulator. It consists of 8 items grouped into 6 non-independent scales: attractiveness, efficiency, perspicuity, dependability, originality, stimulation. Among these, perspicuity, efficiency, and dependability represent pragmatic quality aspects (goal-directed qualities), while stimulation and originality represent hedonic quality aspects (non-goal-directed qualities). Each item consists of a pair of terms with opposite meanings and is rated on a 7-point Likert scale. The NASA-TLX is a validated instrument for subjective workload assessment. It measures perceived task load across six dimensions (mental demand, physical demand, temporal demand, performance, effort, and frustration), each rated on a scale from 0 to 100.

### Data analysis

Trajectory of the virtual representation of the end-effector (x,y,z) was recorded throughout the experiment for off-line analyses (sample frequency: 30 Hz). For each task, the following performance metrics were computed and averaged across repetitions and subjects. The measures we selected have been widely used in simulation-based surgical assessment [20, 21] , as they are valid indicators of surgical technical skill and can predict surgeon’s proficiency [20, 22]. Errors, particularly in precision tasks such as the dexterity exercise, reflects control and coordination ability [22]. Execution time and instruments pathlength inversely correlate with movement efficiency and planning [22], which are critical for reducing operative time and tissue trauma. Further, instruments’ pathlength has been related to surgical experience and practice [23, 24].

#### Incision task

For this task, we analyzed the trajectory in the area corresponding to the predefined cut line. Within this interval, we have computed the following indices: (i) cut length, defined as the cumulative length of tissue cut during the task, computed by summing the portions of the trajectory where collisions between the scalpel and the skin patch occurred; (ii) 3D pathlength, defined as the total Euclidean distance traveled by the scalpel in 3D space during the task, computed over the full trajectory within the cut line interval.

#### Dexterity task

First, we excluded the trajectory points that were outside the skin in the x,y plane. Then, to have comparable trajectories, we have defined as a common starting point, the position where the capsule is 0.5 cm away from the first toroid on the x,y plane. Similarly, the ending point corresponds to the xy position of the capsule when it passes through the last toroid. For this task, the following parameters were analyzed and averaged across repetitions and subjects: (i) 3D path length, as above, representing the total distance traveled by the end-effector; (ii) variability (i.e., standard deviation of the mean) of the pathlength across trials; (iii) execution time, defined as the duration (in seconds) required to complete each trial from start to end; (iv) average depth, calculated as the mean z-position of the end-effector along the trajectory; (v) number of errors, defined as the total number of collisions between the capsule and the toroids. Each collision turned the toroid red and was counted in real time. With four toroids and six repetitions, the maximum number of possible errors was 24.

#### Suture task

Each trial begun when the needle was over the first sphere and finished as it touches the last sphere. Two performance metrics were computed: (i) 3D pathlength, as the total distance covered by the needle holder in 3D space during the task; (ii) execution time, measured as the duration to complete each suture.

All metrics were averaged across repetitions and participants within each group. Where appropriate, standard errors of the mean were also computed.

#### Questionnaires

Both UEQ and NASA TLX have been analyzed using their respective toolboxes. The UEQ toolbox computes mean and standard deviation for each of the six dimensions (see above), separately for the two subject groups. Then, perspicuity, efficiency, and dependability scores are averaged to compute the overall pragmatic quality score, while stimulation and originality are averaged to obtain the hedonic quality score. Similarly, for NASA TLX, mean and standard deviation have been calculated for each of the six workload dimensions (mental demand, physical demand, temporal demand, performance, effort, frustration).

For each subject and index, data exceeding 3 standard deviations from the mean were considered outliers and therefore excluded from the analyses. Performance data between groups (i.e., young, older adults) were compared using Mann-Whitney test, while changes with practice had assessed using Wilcoxon signed-rank test between the first and last repetition, as data deviated from the normal distribution (tested with Shapiro-Wilk test). Results were considered significant with a *p* value < 0.05, Benjamini–Hochberg corrected for multiple comparisons. Effect size of significant results was evaluated using Cohen’s d. All analyses were conducted using MATLAB 2024b and JASP 0.18.3.

## Results

### Pathlength decreased with practice across all participants, while cutting ability increased only in younger individuals

**Fig. 3:**
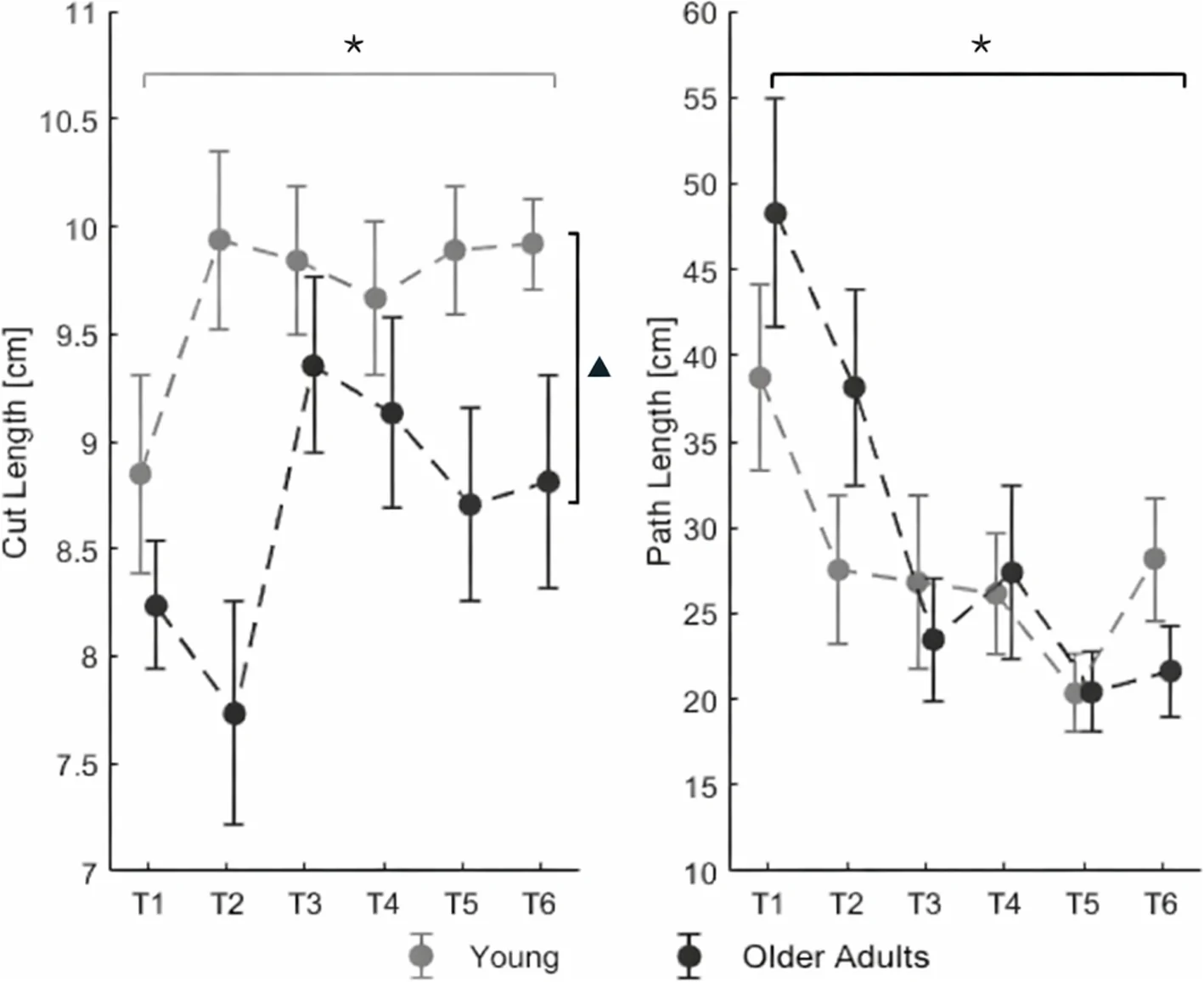
Incision task results. Mean and S.E. of the two groups for the six repetitions. *⋆ * significant difference between first and last repetition (p<.05); *△ * significant difference between groups (p<.05)

As explained above, participants were required to cut the virtual skin following a 11 cm straight line. On average, subjects took (mean ± S.E) 8.8 ± 0.8 s to complete a repetition, with neither difference between groups (Young: 8.3 ± 1.3 s; Old: 9.4 ± 0.9 s), nor improvement with practice (Table 1). However, young subjects were able to cut more tissue than older participants (Young: 9.6 ± 0.2 cm; Old: 8.6 ± 0.3; p=.015; d=0.85; Fig. 3).

**Table 1:** Incision results: mean, standard error (SE), and *p*-values of performance parameters

Parameter	Young (Mean $\boldsymbol{\pm }$ SE)		Older Adults (Mean $\boldsymbol{\pm }$ SE)		*p* (Y vs O)	*p* (T1 vs T6)
	T1	T6	T1	T6		
Duration [s]	10.7 ± 2.2	8.9 ± 1.6	10.0 ± 1.4	9.3 ± 1.7	.305	Y: .478; O: .389
Cut length [cm]	8.9 ± 0.5	9.9 ± 0.2	8.2 ± 0.3	8.8 ± 0.5	.015*	Y: .017*; O: .107
Pathlength [cm]	38.7 ± 5.2	28.2 ± 3.6	48.3 ± 5.9	21.6 ± 2.6	.966	Y: .040; O: .009*
Depth [cm]	0.5 ± 0.2	0.3 ± 0.2	0.1 ± 0.2	0.4 ± 0.2	.361	Y: .351; O: .286

*p-values significant after Benjamini-Hochberg correction

Interestingly, both groups slightly improved their cut length with practice but only the young group reached significance (p=.017; Table 1; Fig. 3). To understand why young subjects were able to cut more tissue than older subjects, we analyzed the mean depth (i.e., the trajectory in the z axis) and the 3D pathlength, which can provide further information on the cutting precision. Both measures did not differ between groups, but pathlength (Fig. 3) was reduced with practice partially in the young group (p=0.040 uncorrected) and significantly in the older adult group (p = .009; Table 1), even though older group’s cut length did not vary. Of notice, by the end of the experiment, the pathlength of the older group was lower than that of the younger group at the beginning of the session (T1 young vs T6 older adults p = 0.022; d = −0.92), and comparable to the performance of the younger group (Fig. 3).

### Younger participants showed better performance in the dexterity task

**Fig. 4:**
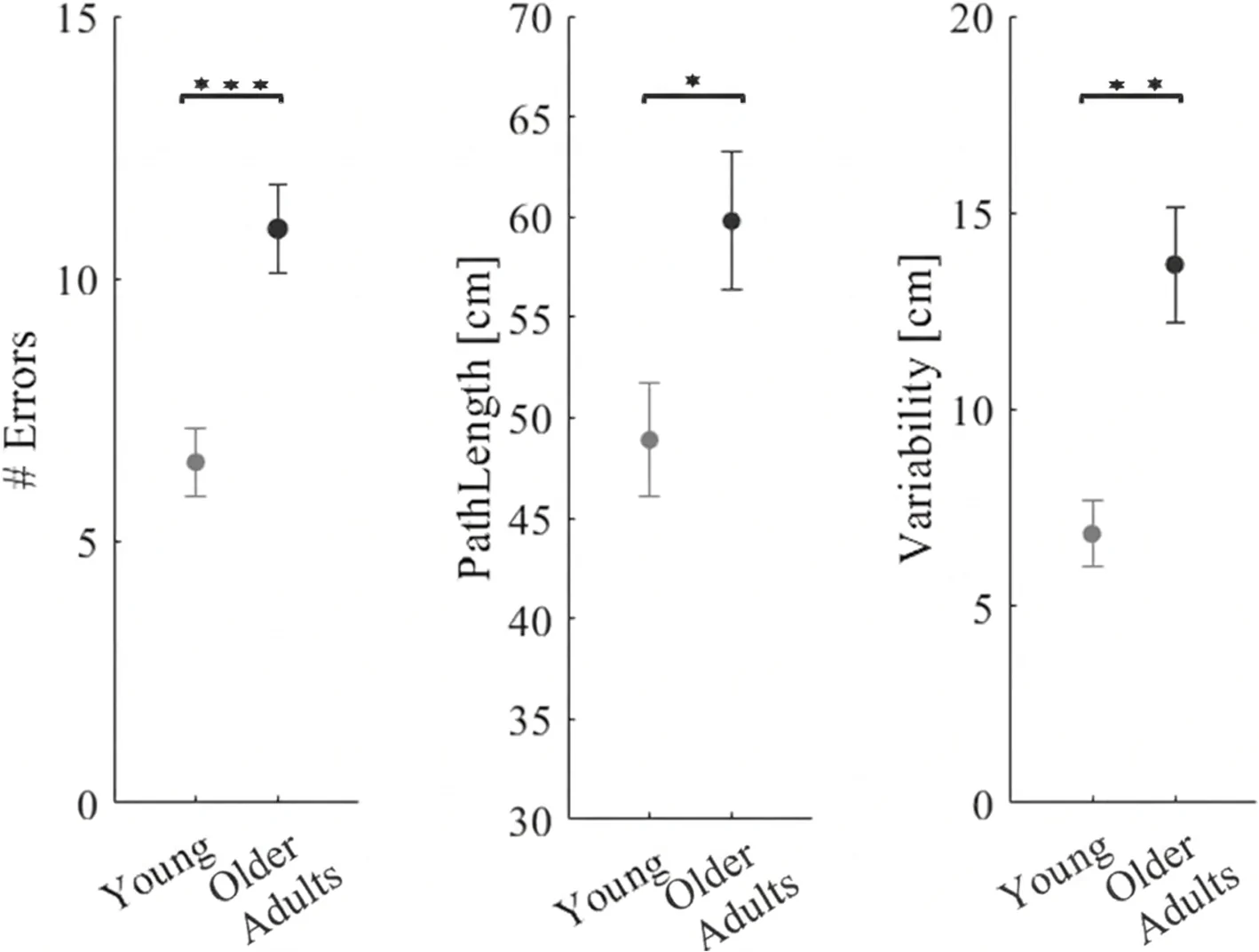
Dexterity Results. Bars indicate S.E. for each group. Left: number of errors (***p<.001); center: mean path length 3D (*p<.05); right: variability of the path length 3D (**p<.01)

As a first step, we have analyzed the errors made by the two groups during the training. Of notice, the maximum numbers of error were 24, which results from having 4 toroids to pass through and 6 repetitions. As summarized in Table 2, younger participants made significantly fewer errors (p=0.001) and exhibited shorter (p=0.032) and less variable (p=0.008) path lengths compared to older participants (Fig. 4). No significant differences were observed in z-position or task completion time.

**Table 2:** Dexterity task performance metrics (mean ± SE) for younger and older participants

Parameter	Young (Mean $\boldsymbol{\pm }$ SE)	Older Adults (Mean $\boldsymbol{\pm }$ SE)	*p*	d
Errors [#]	6.5 ± 1.2	11.0 ± 1.5	.001	-1.30
Path length [cm]	48.9 ± 2.8	59.8 ± 3.4	.032	-0.77
Path variability [cm]	6.8 ± 0.8	13.7 ± 1.5	.008	-1.19

Cohen’s d represents effect size

### Young subjects sutured faster than older participants and improved with practice

**Fig. 5:**
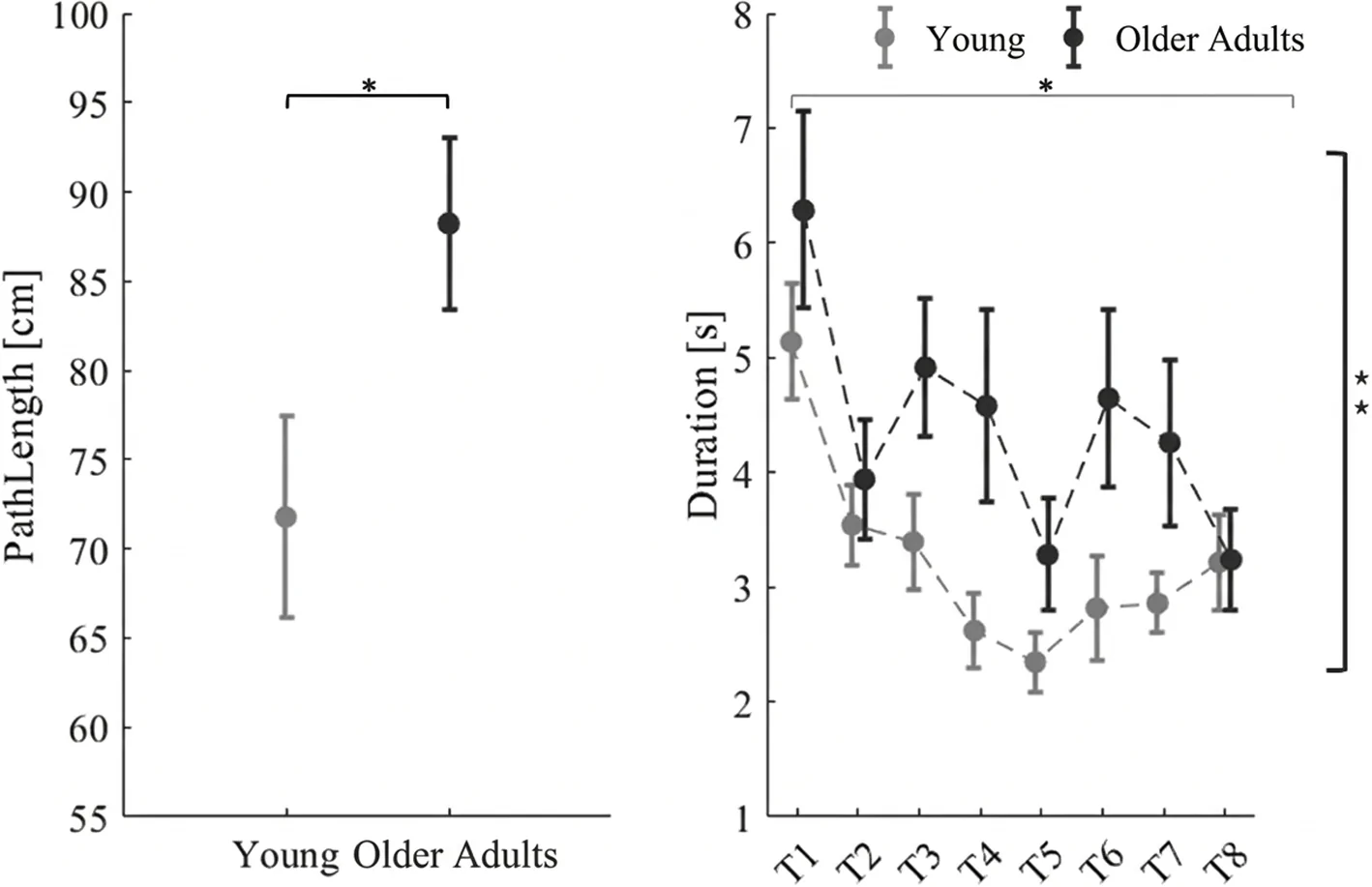
Suture Results. Mean and S.E. for each group. Left: mean path length 3D (young: 71.8 ± 5.7 cm; older adults 88.2 ± 4.8 cm; *⋆ *p<.05); right: trial duration (mean across trials young: 3.2 ± 0.2 s; older adults: 4.4 ± 0.3 s);. *⋆ * significant difference between the first and last trial (p<.05); *△ * group difference (p<.01)

Participants were required to complete a suture guided by spheres on the skin. As performance indices, we measured the time required to complete the task and the 3D pathlength. Both indices were significantly different between the two groups: young individuals were faster (p=.005; d=-1.03) and completed the task with a slightly shorter pathlength (p=.042; d=-0.69) than older adults (Fig. 5). Statistical testing revealed that young participants became significantly faster with practice (p=.010; d=-0.72; Fig. 5). However, visual inspection of the data suggests that older subjects also reduced the execution time, albeit with a higher degree of variability. This is supported by the fact that suturing time in the last repetition was comparable between the two groups (Fig. 5).

### User experience and task load have been evaluated equally by the two groups

Results of the UEQ report positive evaluations for both groups, without significant group differences (Table 3). Values greater than 5 out of 7 have been found for both Pragmatic and Hedonic qualities, that assess simulation’s efficiency and user’s experience, respectively.

**Table 3:** UEQ scores (mean ± SD) for younger and older participants

Scale	Young (Mean $\boldsymbol{\pm }$ SD)	Older Adults (Mean $\boldsymbol{\pm }$ SD)	*p*
Overall UEQ	6.05 ± 0.80	5.95 ± 0.75	.721
Pragmatic Quality	5.85 ± 0.96	5.57 ± 0.92	.341
Hedonic Quality	6.25 ± 0.77	6.36 ± 0.76	.668

The NASA TLX questionnaire results indicate an overall moderate task load for both groups (Table 4). Interestingly, the item with the highest score was related to the mental and perceived effort required to complete the task. Conversely, temporal demand and frustration received the lowest scores (Table 4).

**Table 4:** NASA-TLX scores (mean ± SD) for younger and older participants

Scale	Young (Mean $\boldsymbol{\pm }$ SD)	Older Adults (Mean $\boldsymbol{\pm }$ SD)
Overall	38.9 ± 24.9	47.9 ± 28.1
Mental	41.0 ± 15.5	52.1 ± 26.2
Physical	36.3 ± 24.7	45.5 ± 33.7
Temporal	26.0 ± 24.0	39.2 ± 26.8
Performance	47.8 ± 25.1	55.3 ± 21.4
Effort	55.0 ± 23.3	55.8 ± 30.2
Frustration	30.5 ± 23.1	39.7 ± 27.1

## Discussion

### Aging affects performance using a visuo-haptic surgical simulator

This study aimed to investigate whether performing basic surgical tasks in simulation differ between young and older subjects. Additionally, we wanted to assess if users could improve their performance with practice. Results show that younger outperformed older participants in the incision and dexterity task, and required less time to complete a suture. Notably, while both groups showed some improvement with practice, younger subjects improved more than older ones.

A possible explanation of this results might be that the young cohort improves more than older adults because older adults were already competent. However, the main objective of this study was to investigate the effect of age on performance, regardless of surgical expertise. For this reason, we did not comprise prior surgical experience as an inclusion criterion. However, our sample included participants with surgical background (25% of young and 37% of older adults). Nevertheless, surgical skills is an important factor that should be taken into consideration to enhance the applicability of these findings to the clinical setting. The presence of a proportion of experienced surgeons further strengthens the value of these results. Indeed, aging affects performance in a sample made of both experienced and unexperienced individuals, suggesting that this result is primarily driven by aging rather than previous expertise. The results presented are consistent with prior research on age-related motor decrease. The deterioration of motor performance with aging has been reported in several publications [25], and it has been commonly attributed to neuromuscular degeneration, as well as structural and functional changes in the central and peripheral nervous systems [26]. Such changes result in impaired coordination [27], increased movement variability [28, 29], and slower movement execution [30] in complex motor tasks like surgical ones. In addition, aging affects proprioceptive processing [31], likely contributing to the challenges older adults faced in adapting to the visuo-haptic environment.

Another explanation of these results might be that older adults adopted a more cautious approach, which influenced execution time, path length, and collision rate, that are recognized indicators of surgical proficiency [32–34]. In our study, these indices were higher in the older adult group, suggesting that, irrespective of prior surgical experience, age-related changes in psychomotor performance may contribute to reduced efficiency in simulator-based tasks.

Altogether these results seem to reflect age-related differences in psychomotor performance and learning ability, rather than differences in surgical competence.

### Despite age-related performance differences, the system is equally usable by both groups

To ensure that such group differences were not driven by a possible technology bias [35], participants underwent a familiarization session; moreover, at the end of the experiment, they filled out subjective usability and task load questionnaires. The subjective feedback collected through the UEQ and NASA TLX provides important insight into participants’ experience with the simulator. Both younger and older adults reported high usability scores. This suggests that the system was perceived as efficient, clear, and engaging, regardless of the age of the participants. The NASA TLX revealed a moderate task load in both groups with scores ranging from 26 to 56 out of 100 for all the workload dimensions.

Taken together, these results support the feasibility of using visuo-haptic interfaces, such as the proposed surgical simulator, for both assessment and training across a wide age spectrum. Indeed, the present findings indicate that visuo-haptic simulators, are accessible even to participants with potentially lower digital familiarity, as in the case of older adults. The increasing integration of technology into daily life, combined with the shifting age distribution of the population, might pose a barrier for older adults individuals [36]. In the specific context of surgical training and evaluation, the use of a technologically-enhanced simulator to evaluate technical skills could have introduced a bias that might have underestimated users’ ability. Even though the simulator was highly usable by users belonging to different age groups, future studies should further explore how simulator design can minimize age-related biases, ensuring an optimal training and assessment of older adults surgeons [37].

### Older adults maintain motor learning capacity, though at a slower pace

Both groups demonstrated a partial improvement with practice, indicating that training was generally beneficial in the incision and suturing tasks. Younger subjects appeared to improve at a faster pace than older individuals, who however still maintained the ability to learn. Many studies reported that, whilst motor skills progressively decrease with aging, learning capabilities and performance improvements as a result of training are maintained [38, 39]. In fact, older adults are still capable of motor learning, although often at a slower pace [40, 41]. In our study, we detected a significant pathlength reduction in the incision tasks for the older group, and moderate non-significant improvements in both the incision and suture tasks. A possible explanation for these results is that the older group requires more repetitions to improve performance and reach those of younger individuals. Indeed, studies reported that motor learning seems slower in older adults participants [2], and practice can positively affect motor performance [2, 42]. Nonetheless, some studies have shown that age-related performance gaps can widen with practice [43], suggesting the need for age-specific training strategies. In this context, continuous education should be integrated into surgical practice; specifically, periodic evaluations should be implemented within healthcare systems to detect early signs of performance deterioration and plan personalized retraining protocols to maintain surgical proficiency. To promote surgeons’ engagement, it will be essential to clearly communicate that the purpose of such assessments is not to screen surgeons, but rather to support them by providing objective feedback on psychomotor performance and enabling targeted interventions when necessary. In this respect, the development of targeted and evidence-based training protocols requires further investigation. In particular, it will be crucial to carry out studies aimed to: (i) define optimal performance indices; (ii) assess how these indices vary with practice; (iii) develop evaluation protocols; (iv) determine appropriate retraining programs in case of age-related performance decline.

### Limitations

This study proposes the use of visuo-haptic surgical simulation to train and assess performance decrease due to healthy aging. To fully comprehend the results, it is important to acknowledge that the study presents some limitations that should be addressed in the future to strengthen the present findings.

We have analyzed basic tasks over realistic simulations for three reasons: (i) to isolate and evaluate core motor components such as incision, depth control, and dexterity, which underlie many real surgical procedures [44]; (ii) because there is evidence that gesture-level and maneuver-level metrics are predictive of global surgical performance [22, 44]; (iii) to avoid biases related to task complexity, which involves both technical and procedural skills and is affected by a high degree of variability. Although basic exercises, when properly instrumented and analyzed, can provide meaningful insights into complex skill development [22], performance on realistic simulations might reveal additional information about experienced surgeons’ behaviors. In this context, future studies should replicate the present findings with more complex simulations, as well as real-world surgical procedures.

Moreover, we tested subjects with different backgrounds, as we wanted to assess if performance decreases with age regardless of one’s surgical background; Although some participants had surgical training, we did not stratify or analyze the data based on surgical experience. For this reason, future studies should include dedicated comparisons across experience levels to investigate how professional expertise may compensate for age-related motor decrease.

Other limitations of the study are the lack of information on participants’ proficiency in manual activities, such as musical instrument practice, video gaming, skilled manual trades, that could have potentially influenced the results. Also, we have enrolled people aged 20-40 and 50-70, corresponding to the young and older group, respectively. However, the precise onset of motor decrease remains unclear. Future work should be focused on longitudinal studies that can provide more definitive insights into the onset and progression of age-related performance decrease. Due to the broad age ranges that we considered, it is likely that the older group in our study included individuals who had not yet experienced significant performance decrease being aged 50 to 60. Similarly, the performance decrease of a 50-year-old might be smaller than that of a 70-year-old. Having tested two broad groups may have led to an underestimation of the impact of aging on participants’ performance. Presumably, a more homogeneous sample might reveal stronger effects of age and practice-related improvements.

Finally, one could argue that the system we proposed might not not fully reflect real-world surgical skills and, therefore transferability might be limited. However, surgical simulators are established tools for training and assessment, and studies demonstrated skills transfer to the operative setting [45, 46]. In particular, the system provides haptic feedback that plays a key role in surgical practice, as it increases task accuracy and ultimately improves transferability [47]. Nevertheless, to develop a system able to quantitatively assess manual skills in aging surgeons, it will be crucial to carry out studies aimed at establishing construct, concurrent, and predictive validity of the simulator.

## Conclusion

The present study supports the idea that visuo-haptic simulation can serve as a basis for evaluating aging surgeons by providing objective performance metrics. Results show that younger and older participants perform basic surgical skills differently, suggesting that the simulator that we have designed might be a valuable tool to assess surgeons’ residual abilities. Further, it could be an effective training tool to counteract age-related performance decrease. In this context, it would be important to carefully design simulations and define the amount of practice required to ensure that aging surgeons maintain proficiency in surgical tasks. Future studies will investigate whether these findings also apply to simulated surgeries involving multiple abilities simultaneously. Moreover, by collecting data from people of different ages, it would be possible to identify the age at which motor performance begins to decrease. Understanding when and how surgical performance starts to decrease is crucial to develop continuous education programs aimed at maintaining surgical skills and ultimately ensure patients safety.

## Figures and Tables

**Fig. 6:**
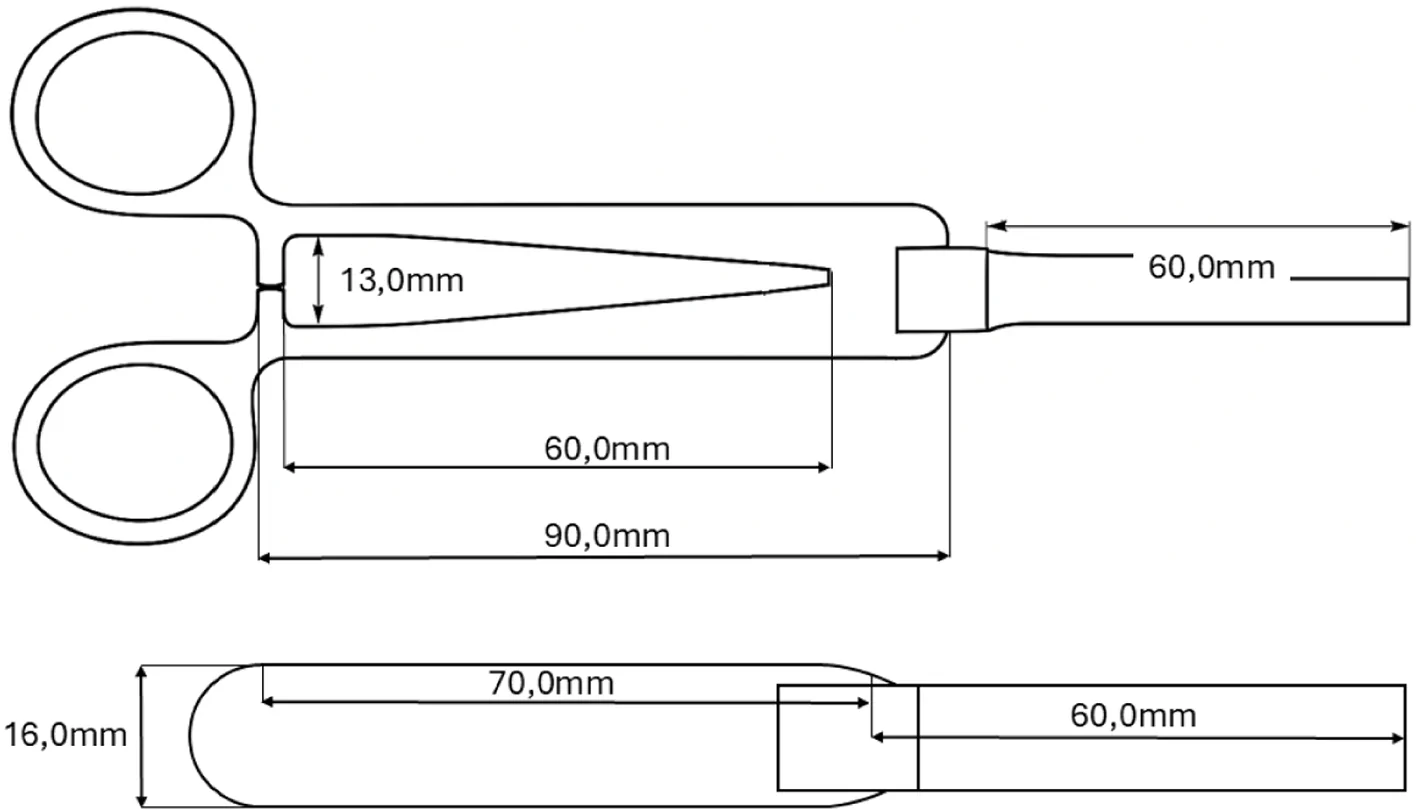
Physical handle. Top: needle holder CAD printed for the suture task. Bottom: scalpel handle CAD printed for the incision task

## Data Availability

The datasets used and analysed during the current study are available from the corresponding author on reasonable request

## Appendix A: Supplementary Methods

### Hardware

The Geomagic Touch has three actuated joints, which define the position of the end-effector, and three under-actuated joints that determine its orientation [48], resulting into 6 Degrees of Freedom. When the virtual position of the end effector collides with a virtual object, a force is generated depending to the object’s stiffness. The device’s specifications are as follows: workspace dimensions of 43x35x17 cm, maximum force output 3.3 N and stiffness values of X > 1.26 N/mm, Y axis > 2.31 N/mm, Z axis > 1.02 N/mm [11].

The end-effector of the haptic device has been customized to make it more similar to real surgical tools. By modeling and 3D-printing surgical instrument handles, we have created two hand effectors: A scalpel handle, for the incision task, and a needle holder (Fig. 6) designed for suturing. Additionally, the device’s standard stylus handler could be used for the exercise not requiring any surgical tool (i.e., the dexterity exercise).

### Software

In SOFA, virtual objects are composed of three interconnected models: (i) behavior, that describes the internal mechanical behavior of an object; (ii) collision, handling the interaction between objects; (iii) visual, which implements the graphical rendering of the objects. These models are interconnected through mappings to ensure continuity and coherency when perturbations occur; the collision model and behavior model are linked via a topological mapping, while the visual model typically uses an identity mapping to reflect the collision geometry.

For this project, we used SOFA version 20.12.02, and two plugins: the Geomagic plugin (provided by 3D Systems, OpenHaptics SDK), and SofaPython3 The former was used to interface the simulation with the haptic device, enabling real-time force feedback during interactions; the latter, that requires Python 3.7.7, allows for scripting within SOFA using a Python interpreter.

### Skin model

In our project, we have implemented a virtual model of the skin having the following parameters: Young Modulus = 4 kPa; Poisson’s Ratio = 0.1; Density = 2.0 g/cm$^2$ [49]. The behavior model was defined as a box with a tetrahedral internal structure, simulating the elasticity and deformation of human skin. The collision model was generated by mapping the tetrahedral structure to triangles using a topological mapping, and the visual model was created as an identity mapping from the collision model to ensure accurate rendering.

## Abbreviations

EHQ: Edinburgh Handness Questionnaire
Y: Young
O: Older Adults
ROI: Region of Interest
SE: Standard Error
STD: Standard Deviation
TLX: Task Load Index
UEQ: User Experience Questionnaire
VE: Virtual Environment
